# ﻿Six new species of the leafhopper subgenus *Pediopsoides* (*Pediopsoides*) (Hemiptera, Cicadellidae, Eurymelinae, Macropsini) from China

**DOI:** 10.3897/zookeys.1165.81776

**Published:** 2023-06-05

**Authors:** Hu Li, Juan Li, Michael D. Webb, Jia-Jia Wang, Ren-Huai Dai

**Affiliations:** 1 Shaanxi Key Laboratory of Bio-resources, School of Biological Science & Engineering, Shaanxi University of Technology; Qinling-Bashan Mountains Bioresources Comprehensive Development C.I.C.; State Key Laboratory of Biological Resources and Ecological Environment of Qinling-Bashan, Hanzhong, Shaanxi, 723000 China Institute of Entomology of Guizhou University Guiyang China; 2 Institute of Entomology of Guizhou University, The Provincial Key Laboratory for Agricultural Pest Management of Mountainous Region, Guiyang, Guizhou, 550025 China Shaanxi University of Technology Hanzhong China; 3 Xi’an Zhongtie Middle School, Xi’an, Shaanxi, 710054 China Xi’an Zhongtie Middle School Xi’an China; 4 The Natural History Museum, London, SW7 5BD UK The Natural History Museum London United Kingdom; 5 College of Biology and Food Engineering, Chuzhou University, Chuzhou, Anhui, 239000 China Chuzhou University Chuzhou China

**Keywords:** Auchenorrhyncha, distribution, Homoptera, macropsine, morphology

## Abstract

The nominate subgenus Pediopsoides (Pediopsoides) Matsumura, 1912 is widely distributed in the Oriental region but has high species diversity only in southern China. The present paper describes and illustrates six new species of Pediopsoides (Pediopsoides) namely, P. (P.) ailaoshanensis Li & Dai, **sp. nov.**, P. (P.) quadrispinosus Li & Dai, **sp. nov.**, P. (P.) flavus Li & Dai, **sp. nov.**, and P. (P.) pianmaensis Li & Dai, **sp. nov.** all collected in Yunnan Province, southwestern China, P. (P.) maoershanensis Li & Dai, **sp. nov.**, found in Guangxi Autonomous Region, southern China, and P. (P.) huangi Li & Dai **sp. nov.**, from Taiwan, previously incorrectly recorded as a new name by Li & Dai, 2018 in Dai et al. 2018: 203 for P. (P.) femorata Huang & Viraktamath, 1993 (nec *Pediopsisfemorata* Hamilton, 1980). Two new junior synonyms of *Sispocnis* Anufriev, 1967 are proposed, i.e., *Digitalis* Liu & Zhang, 2002, **syn. nov.** and *Neosispocnis* Dmitriev, 2020, **syn. nov.**

## ﻿Introduction

**Figures 1–11.**
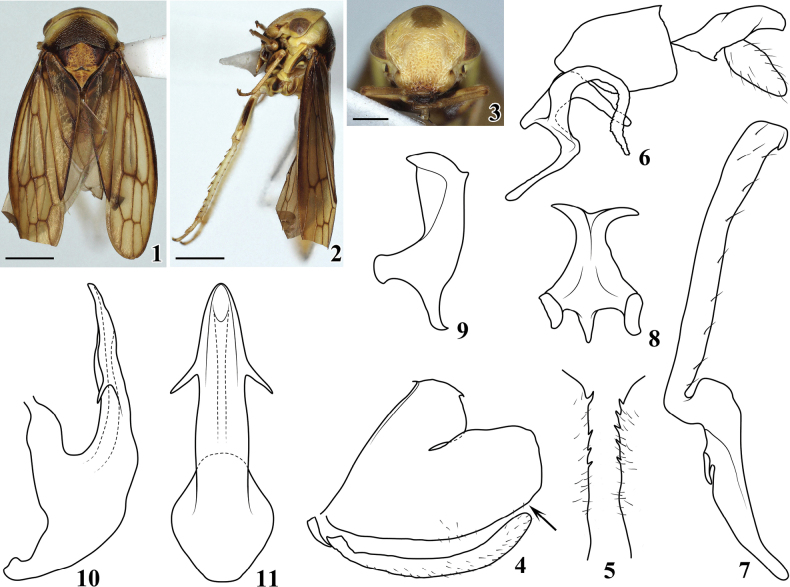
Pediopsoides (Pediopsoides) ailaoshanensis Li & Dai, sp. nov. **1** male habitus, dorsal view **2** male habitus, lateral view **3** face **4** male pygofer and subgenital plate, lateral view **5** pygofer inner ventral distal margins in direction of arrow in Fig. 4, ventral view **6** dorsal connective and 10^th^ tergite, lateral view **7** style, lateral view **8** connective, dorsal view **9** connective, lateral view **10** aedeagus, lateral view **11** aedeagus, ventral view. Scale bars: 1 mm (**1, 2**); 0.5 mm (**3**).

**Figures 12–22.**
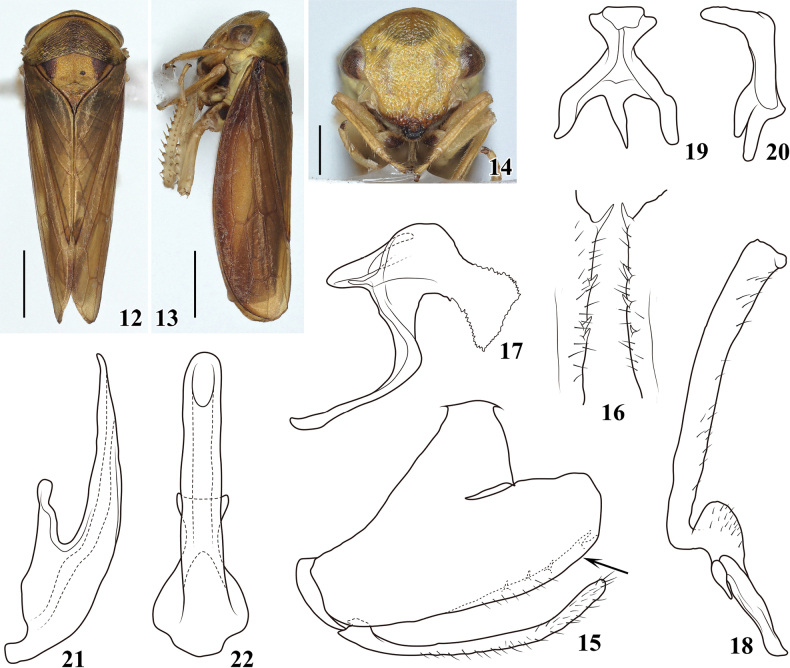
Pediopsoides (Pediopsoides) maoershanensis Li & Dai, sp. nov. **12** male habitus, dorsal view **13** male habitus, lateral view **14** face **15** male pygofer and subgenital plate, lateral view **16** pygofer inner ventral distal margins in direction of arrow in Fig. 15, ventral view **17** dorsal connective, lateral view **18** style, lateral view **19** connective, dorsal view **20** connective, lateral view **21** aedeagus, lateral view **22** aedeagus, ventral view. Scale bars: 1 mm (**12, 13**); 0.5 mm (**14**).

**Figures 23–33.**
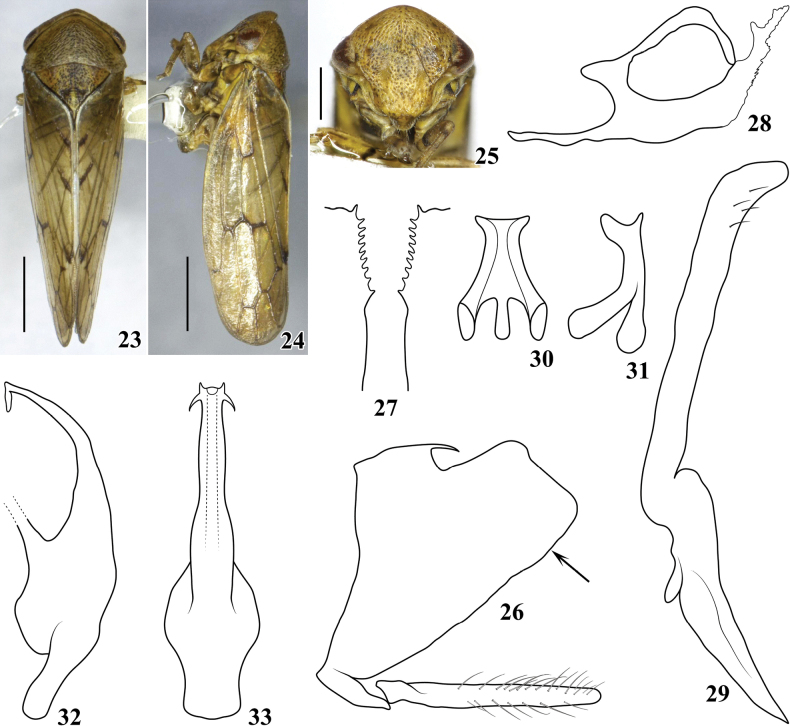
Pediopsoides (Pediopsoides) quadrispinosus Li & Dai, sp. nov. **23** male habitus, dorsal view **24** male habitus, lateral view **25** face **26** male pygofer and subgenital plate, lateral view **27** pygofer inner ventral distal margins in direction of arrow in Fig. 26, ventral view **28** dorsal connective, lateral view **29** style, lateral view **30** connective, dorsal view **31** connective, lateral view **32** aedeagus, lateral view **33** aedeagus, ventral view. Scale bars: 1 mm (**23, 24**); 0.5 mm (**25**).

**Figures 34–43.**
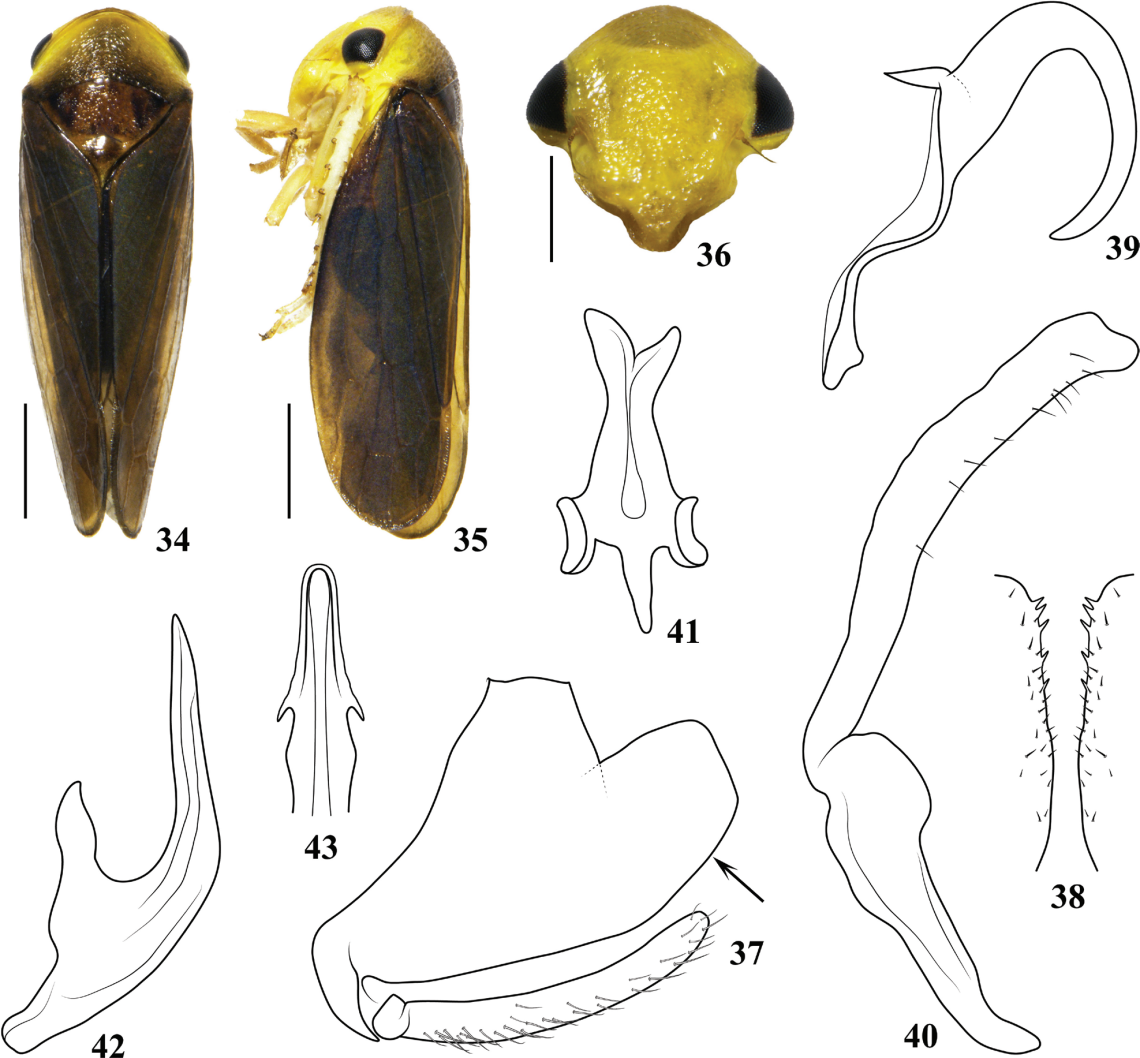
Pediopsoides (Pediopsoides) flavus Li & Dai, sp. nov. **34** male habitus, dorsal view **35** male habitus, lateral view **36** face **37** male pygofer and subgenital plate, lateral view **38** pygofer inner ventral distal margins in direction of arrow in Fig. 37, ventral view **39** dorsal connective, lateral view **40** style, lateral view **41** connective, dorsal view **42** aedeagus, lateral view **43** apical half of aedeagus, ventral view. Scale bars: 1 mm (**34, 35**); 0.5 mm (**36**).

**Figures 44–53.**
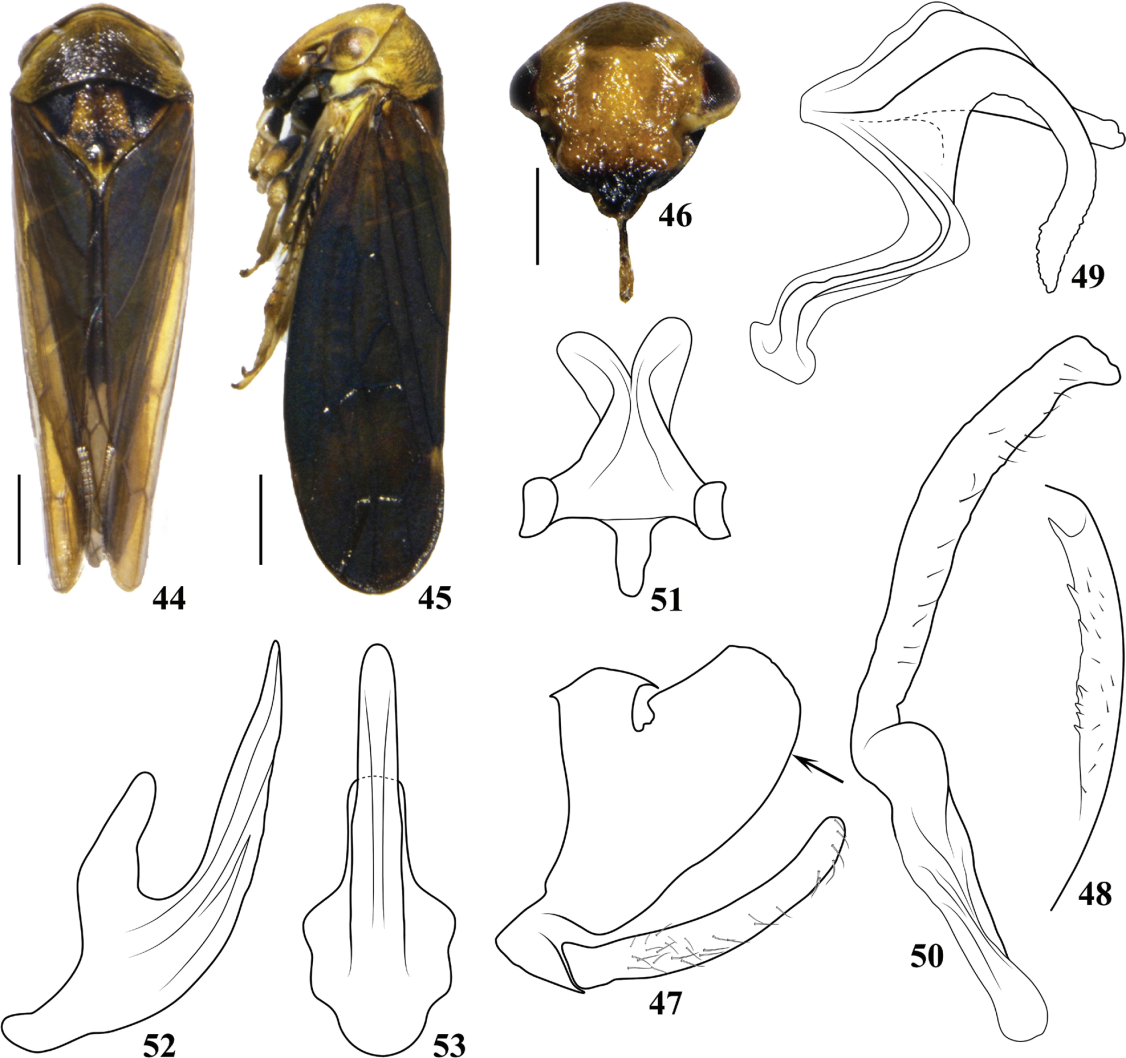
Pediopsoides (Pediopsoides) pianmaensis Li & Dai, sp. nov. **44** male habitus, dorsal view **45** male habitus, lateral view **46** face **47** male pygofer and subgenital plate, lateral view **48** pygofer inner ventral distal margin in direction of arrow in Fig. 47, ventromedial view **49** dorsal connective, lateral view **50** style, lateral view **51** connective, dorsal view **52** aedeagus, lateral view **53** aedeagus, ventral view. Scale bars: 1 mm (**44, 45**); 0.5 mm (**46**).

*Pediopsoides* Matsumura, 1912 is a relatively small genus in the arboreal leafhopper tribe Macropsini (Eurymelinae). Within Macropsini, *Pediopsoides* is recognized by the weak striations on the pronotum (Figs 1, 12, 23, 34, 44), and posterior margins of male pygofer usually with short, inturned spines (Figs 5, 16, 27, 38, 48) (Hamilton 1980); it is distributed in all zoogeographical regions except in the Australian and Neotropical regions.

Hamilton (1980) revised the genus and divided it into five subgenera of which Pediopsoides (Celopsis) Hamilton, 1980 was raised to generic level by Dai et al. (2018). Of the remaining four subgenera, P. (Nanopsis) Freytag, 1974 (currently P. (Pseudonanopsis) Dmitriev, 2020) is distributed in the Nearctic Region and P. (Kiamoncopsis) Linnavuori, 1978 is distributed in the Afrotropical region. The remaining two subgenera, P. (Pediopsoides) and P. (Sispocnis) Anufriev, 1967 (following Li et al. 2019, not Dmitriev 2020, see below) are distributed mainly in China (see checklist). As Dmitriev (2020: 38) overlooked the earlier type species designation of *Sispocnis* by Li et al. (2019), his actions are invalid (ICZN 1999, Art. 70.2) and *Neosispocnis* Dmitriev, 2020 should be treated as a junior subjective synonym of *Sispocnis*, syn. nov. In addition, Dai and Zhang (2009: 23) synonymized *Digitalis* Liu & Zhang, 2002 with *Pediopsoides* but Dai and Zhang (2009: 28) also synonymized the type species of *Digitalis* (*D.striolatus* Liu & Zhang, 2002) with P. (Sispocnis) aomians (Kuoh, 1981), therefore *Digitalis* should also be treated as a junior subjective synonym of *Sispocnis*, syn. nov. and not of *Pediopsoides*.

According to the known host records, *Pediopsoides* feeds on species of *Juglans* (Juglandaceae) and willows (Tishechkin 2016).

Pediopsoides (Pediopsoides) includes 13 species at present, of which 11 are known from China, mainly southern China (see Checklist). It is the largest subgenus within the genus *Pediopsoides* and is characterized by the following features: pronotum with oblique striations, forewing with two ante-apical cells, dorsal connective armed only at apex and freely attached to tenth tergite, side of male pygofer without an articulated lobe and male pygofer process bifid or with fine teeth (Hamilton 1980; Li et al. 2016).

In this paper six new species of the nominate subgenus from southern China are described and illustrated.

## ﻿Materials and methods

The higher classification system and morphological terminology used in this work follow Hamilton (1980) and Dietrich and Thomas (2018). The specimens were collected by general sweeping. External morphology was observed under an Olympus SZX7 and an Olympus BX43 microscopes. The habitus images of adults were obtained by using a KEYENCE VHX-1000 system for Pediopsoides (Pediopsoides) quadrispinosus Li & Dai, sp. nov., P. (P.) flavus Li & Dai, sp. nov. and P. (P.) pianmaensis Li & Dai, sp. nov., and a KEYENCE VHX-7000 system for P. (P.) ailaoshanensis Li & Dai, sp. nov. and P. (P.) maoershanensis Li & Dai, sp. nov. Male genitalia drawings were prepared and edited utilizing Adobe Illustrator CS6 and Photoshop CS6. The body length was measured from the apex of the head to the folded forewings and is given in millimeters (mm).

The material examined is deposited in the Museum of Zoology and Botany, Shaanxi University of Technology, Hanzhong, China (**SUHC**), and the Institute of Entomology of Guizhou University, Guiyang, China (**GUGC**).

## ﻿Taxonomy

**Genus *Pediopsoides* Matsumura, 1912**

### ﻿Subgenus Pediopsoides (Pediopsoides)

Taxon classificationAnimaliaHemipteraCicadellidae

Matsumura, 1912

11CDDD63-0152-5F22-AFB0-035D3ACF6012

Pediopsoides Matsumura, 1912: 305.
Pediopsoides (Pediopsoides) : Hamilton, 1980: 896.

#### Type species.

*Pediopsoidesformosanus* Matsumura, 1912, by original designation.

#### Distribution.

Oriental region: China, Japan, and India.

### Checklist to species of the subgenus

Pediopsoides (Pediopsoides) Matsumura

**Pediopsoides (Pediopsoides) ailaoshanensis Li & Dai, sp. nov. (Figs 1–11)**

**Distribution.** China (Yunnan Prov.).

**Pediopsoides (Pediopsoides) albus Li, Dai & Li (Figs 61, 62)**

P. (P.) alba Li, Dai & Li, 2016: 342; figs 1–3, 10–19.

P. (P.) albus, Lemaître et al., 2017: 677.

**Distribution.** China (Yunnan Prov.).

**Pediopsoides (Pediopsoides) amplificatus Li, Dai & Li (Figs 63, 64)**

P. (P.) amplificata Li, Dai & Li, 2016: 344; figs 4–6, 20–27.

P. (P.) amplificatus, Lemaître et al., 2017: 677.

**Distribution.** China (Guangdong Prov.).

**Pediopsoides (Pediopsoides) anchorides Yang & Zhang (Figs 65, 66)**

P. (P.) anchorides Yang & Zhang, 2013: 585; figs 1E–H, 3A–H.

**Distribution.** China (Yunnan Prov.).

**Pediopsoides (Pediopsoides) bispinatus Li, Dai & Li (Figs 67, 68)**

P. (P.) bispinata Li, Dai & Li, 2012: 539; figs 1–4, 8–17.

P. (P.) bispinatus, Lemaître et al., 2017: 677.

**Distribution.** China (Guangxi Prov.).

**Pediopsoides (Pediopsoides) damingshanensis Li, Dai & Li (Figs 69, 70)**

P. (P.) damingshanensis Li, Dai & Li, 2013: 17; figs 1–3, 7–13.

**Distribution.** China (Guangxi Prov.).

**Pediopsoides (Pediopsoides) flavus Li & Dai, sp. nov. (Figs 34–43)**

**Distribution.** China (Yunnan Prov.).

**Pediopsoides (Pediopsoides) formosanus Matsumura**

*Pediopsoidesformosanus* Matsumura, 1912: 306.

P. (P.) formosanus, Hamilton, 1980: 896.

**Distribution.** China (Taiwan).

**Figures 54–60.**
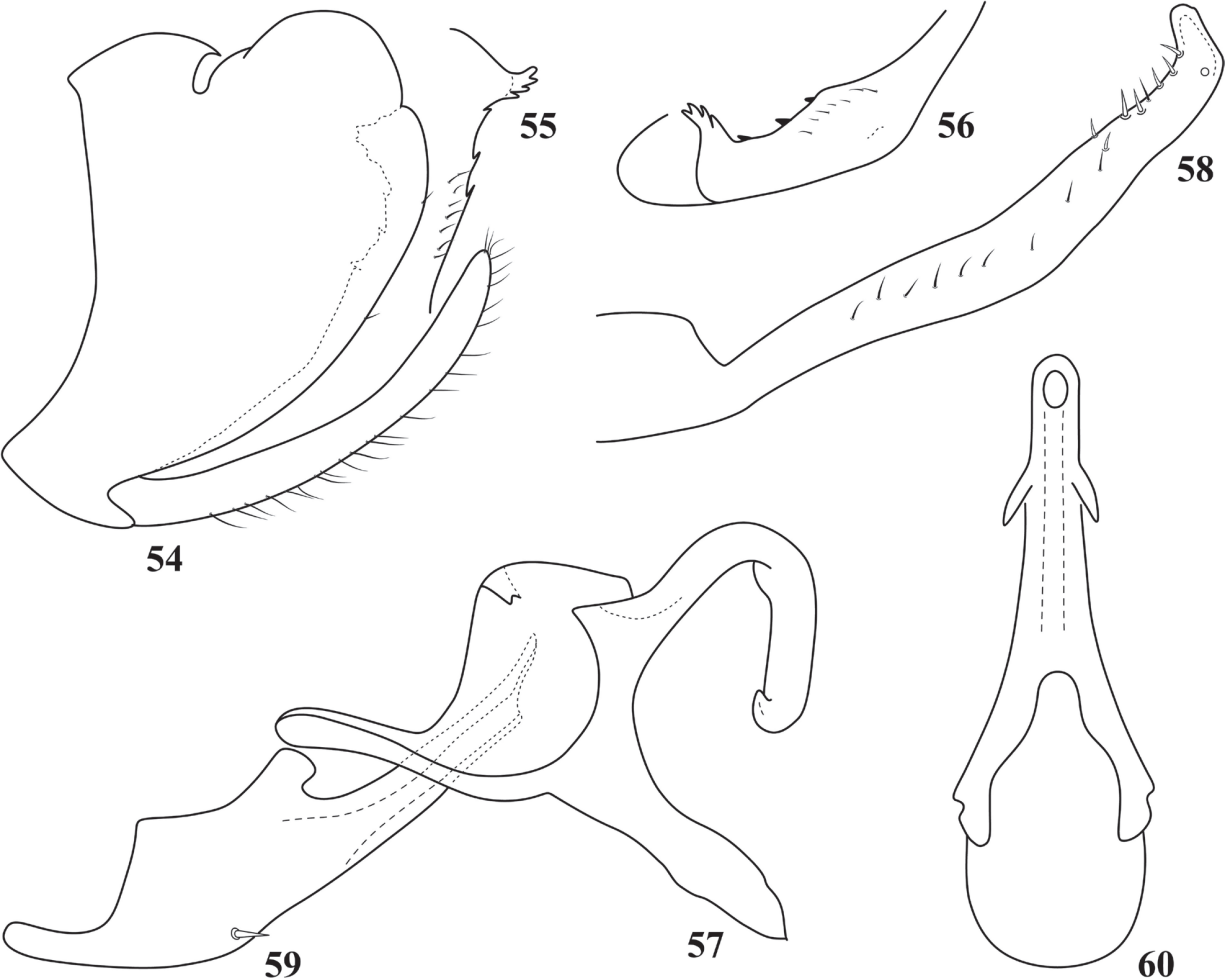
Pediopsoides (Pediopsoides) huangi Li & Dai, sp. nov. (after Huang and Viraktamath 1993) **54** male pygofer and subgenital plate, lateral view **55** pygofer inner ventral distal margin, ventral view **56** pygofer inner ventral distal margin, ventromedial view **57** dorsal connective, lateral view **58** style, lateral view **59** aedeagus, lateral view **60** aedeagus, dorsal view.

**Pediopsoides (Pediopsoides) huangi Li & Dai, sp. nov. (Figs 54–60)**

P. (P.) femorata Huang & Viraktamath, 1993: 365; figs 18–28, misapplication.

P. (P.) huangi Li & Dai, 2018 in Dai et al. 2018: 203. Erroneously treated as a new name for P. (P.) femorata Huang & Viraktamath, 1993 (nec *Pediopsisfemorata* Hamilton, 1980).

**Distribution.** China (Taiwan).

**Pediopsoides (Pediopsoides) jingdongensis Zhang (Figs 71, 72)**

P. (P.) jingdongensis Zhang, 2010: 58; figs 5–8, 21–31.

**Distribution.** China (Yunnan Prov.).

**Pediopsoides (Pediopsoides) kodaianus Viraktamath (Figs 73, 74)**

P. (P.) kodaiana Viraktamath, 1996: 188; figs 25–36.

P. (P.) kodaianus, Lemaître et al., 2017: 677.

**Distribution.** India (Tamil Nadu).

**Pediopsoides (Pediopsoides) longiapophysis Li, Dai & Li (Figs 75, 76)**

P. (P.) longiapophysis Li, Dai & Li, 2016: 346; figs 7–9, 28–35.

**Distribution.** China (Guangdong Prov.).

**Pediopsoides (Pediopsoides) maoershanensis Li & Dai, sp. nov. (Figs 12–22)**

**Distribution.** China (Guangxi Prov.).

**Pediopsoides (Pediopsoides) nigrolabium Li, Dai & Li (Figs 77, 78)**

P. (P.) nigrolabium Li, Dai & Li, 2012: 540; figs 5–7, 18–27.

**Distribution.** China (Guangxi Prov.).

**Pediopsoides (Pediopsoides) pianmaensis Li & Dai, sp. nov. (Figs 44–53)**

**Distribution.** China (Yunnan Prov.).

**Pediopsoides (Pediopsoides) quadrispinosus Li & Dai, sp. nov. (Figs 23–33)**

**Distribution.** China (Yunnan Prov.).

**Pediopsoides (Pediopsoides) satsumensis (Matsumura) (Figs 79, 80)**

*Pediopsissatsumensis* Matsumura, 1912: 311.

P. (P.) satsumensis, Hamilton, 1980: 896; fig. 71.

**Distribution.** Japan (Kyushu).

**Pediopsoides (Pediopsoides) tishetshkini Li, Dai & Li (Figs 81, 82)**

P. (P.) tishetshkini Li, Dai & Li, 2013: 18; figs 4–6, 14–22.

**Distribution.** China (Guangxi Prov.).

### ﻿Key to species of the subgenus Pediopsoides (Pediopsoides) Matsumura (China, except where indicated) (males)

Pediopsoides (Pediopsoides) formosanus (Matsumura) from Taiwan is excluded from the key since it is known only from the female.

**Table:** 

1	Aedeagal shaft in ventral view terminally with a lamelliform triangular shaped expansion on each side (Fig. 64)	**P. (P.) amplificatus**
–	Aedeagal shaft in ventral view gradually tapered to round or acute apex	**2**
2	Aedeagus with pair of processes at apex	**3**
–	Aedeagus with processes absent or if present not at apex	**4**
3	Aedeagal shaft with pair of slender apical processes twisted ventrally (Figs 71, 72)	**P. (P.) jingdongensis**
–	Aedeagal shaft with two pairs of spine-like processes apically, directed laterally (Figs 32, 33)	**P. (P.) quadrispinosus sp. nov.**
4	Aedeagal shaft relatively broad in lateral view, with pair of processes at base (Figs 61, 62); pygofer side with relatively broad biolobed processes caudoventrally (Li et al. 2016: figs 10–11)	**P. (P.) albus**
–	Aedeagal shaft narrow in lateral view, without processes or if present not at base; pygofer side with minute branched processes or fine teeth caudoventrally	**5**
5	Aedeagal shaft with distinct pair of spine-like processes near apex or at midlength, laterally	**6**
–	Aedeagal shaft without processes	**9**
6	Aedeagal shaft processes near apex, relatively small and weakly sclerotized (Fig. 43); dorsal connective with one simple long apical process tapered gradually and twisted basally (Fig. 39)	**P. (P.) flavus sp. nov.**
–	Aedeagal shaft processes nearly at midlength, well sclerotized	**7**
7	Aedeagal shaft processes directed dorsally (Figs 73, 74). India	**P. (P.) kodaianus**
–	Aedeagal shaft processes directed ventrally basally	**8**
8	Dorsal connective with additional process from ventral margin straight and pointed ventrocaudally (Fig. 57); pygofer with apical half of ventral margin expanded inward with several teeth (Figs 55, 56)	**P. (P.) huangi sp. nov.**
–	Dorsal connective with additional process from ventral margin long, strongly sinuate, and twisted ventrally (Fig. 6); pygofer with apical half of ventral margin having four or five small teeth (Fig. 4)	**P. (P.) ailaoshanensis sp. nov.**
9	Style with apex bearing a spine-like process (Li et al. 2013: fig. 16); dorsal connective centrally produced into bifurcate process (Li et al. 2013: fig. 21)	**P. (P.) tishetshkini**
–	Style without process; dorsal connective without bifurcate process	**10**
10	Pygofer ventral margin with single process widened at base and slanting inwards (Yang and Zhang 2013: figs 3A, 3H); aedeagal shaft abruptly bent dorsally at apical 1/3 (Fig. 66)	**P. (P.) anchorides**
–	Pygofer ventral margin multifid in general; aedeagal shaft gradually bent dorsally	**11**
11	Dorsal connective stout and axe-like in lateral view with apical half wide with serrated margins (Fig. 17)	**P. (P.) maoershanensis sp. nov.**
–	Dorsal connective not as above (Fig. 17)	**12**
12	Dorsal connective with one additional process centrally	**13**
–	Dorsal connective without additional process centrally	**15**
13	Dorsal connective as in Fig. 49; pygofer ventral margin with apical half with prominent tooth-like distal process preceded by a series of small teeth (Fig. 48)	**P. (P.) pianmaensis sp. nov.**
–	Dorsal connective and pygofer not as above	**14**
14	Aedeagal shaft with round apex in lateral view (Fig. 75); pygofer with few minute teeth at caudoventral corner (Li et al. 2016: fig. 29); dorsal connective strongly developed, with additional process long and twisted caudaoventrad (Li et al. 2016: fig. 31)	**P. (P.) longiapophysis**
–	Aedeagal shaft with acute apex in lateral view (Fig. 77); pygofer with branched process at caudoventral corner and several small teeth on distal half of ventral margin (Li et al. 2012: figs 20–21); dorsal connective weakly developed, with additional process short and pointed caudad (Li et al. 2012: fig. 25)	**P. (P.) nigrolabium**
15	Pygofer ventral margin with inflexed bifurcate processes (Hamilton 1980: fig. 71); dorsal connective stout S-shaped (Hamilton 1980: fig. 71). Japan	**P. (P.) satsumensis**
–	Pygofer ventral margin slightly serrated caudally or with two small spine-like processes; dorsal connective slender S-shaped or bent at midlength	**16**
16	Pygofer ventral margin slightly serrated (Li et al. 2013: fig. 7); dorsal connective relatively well developed, S-shaped (Li et al. 2013: fig. 13)	**P. (P.) damingshanensis**
–	Pygofer ventral margin with two small spine-like processes (Li et al. 2012: figs 10–11); dorsal connective weakly developed, abruptly bent at midlength (Li et al. 2012: fig. 15)	**P. (P.) bispinatus**

### ﻿Species descriptions

#### ﻿ Pediopsoides (Pediopsoides) ailaoshanensis

Taxon classificationAnimaliaHemipteraCicadellidae

Li & Dai sp. nov.

2FD8B2D8-F38F-5013-8DF8-121816328D19

https://zoobank.org/4C853E55-036C-4C9E-BA7B-C43EFC953697

Figs 1–11

##### Material examined.

***Holotype*** ♂, China: Yunnan Province, Yuxi City, Ailaoshan National Natural Reserve, 2400 m above sea level, 08.viii.2015, collected by Yun-Fei Wu and Jia-Jia Wang (SUHC).

##### Description.

***Body color*** (Figs 1–3). Dorsum yellowish to brown. Head (Fig. 1) yellowish, frontal margin of central part pale brown; face (Fig. 3) evenly yellowish except postclypeus with large pale brown spot centrally and anteclypeus brown to black; eyes brown with reddish tinge. Pronotum (Fig. 1) yellowish adjacent to eyes, other parts dark brown, posterior margin nearly black. Mesonotum (Fig. 1) yellow-brown, basal triangles dark brown, striations, and punctures on surface darker brown. Forewings (Figs 1, 2) pale brown, veins darker brown. Legs yellowish with brown markings.

***Body morphology*** (Figs 1–3). Head including eyes (Fig. 1) narrower than pronotum, crown short, parallel sided. Face across eyes (Fig. 3) wider than long, surface with clear punctures and stripes, distance between ocelli nearly 8.2 × that from ocellus to adjacent eye. Pronotum (Fig. 1) broad, 2.5 × wider than long, with almost transverse striations, anterior margin convex and posterior margin concave at midlength. Mesonotum (Fig. 1) ~ 1.5 × longer than pronotum. Forewing (Fig. 1) with veins prominent.

***Male genitalia*** (Figs 4–11). Pygofer side (Fig. 4) broad basally, lobe stout with truncated caudal margin, ventral margin with a few scattered setae, distal half with four or five small teeth (Fig. 5). Subgenital plate (Fig. 4) slender, shorter than ventral margin of pygofer, surface with fine setae. Dorsal connective (Fig. 6) strongly developed, S-shaped, with median long slender process directly mesally from ventral margin, apical half strongly curved ventrally and twisted. Style (Fig. 7) angled at basal 2/5. Connective (Figs 8, 9) with anterior margin wider than posterior margin, and both lateral arms twisted dorsally. Aedeagus (Figs 10, 11) broad basally, tapered to acute apex, shaft almost straight in lateral view, with pair of spine-like processes from lateral margins at midlength, dorsal apodeme developed but short, preatrium broad, gonopore subapical to apical on ventral surface.

##### Measurement.

Body length (including tegmen): 5.1 mm.

##### Distribution.

China (Yunnan Province).

##### Etymology.

The specific epithet refers to the type locality of the new species, Ailaoshan National Natural Reserve (Yunnan Province), combined with the Latin adjectival suffix -*ensis*, meaning from a place.

##### Remarks.

The body appearance and color pattern of the new species is similar to several other congeners especially P. (P.) pianmaensis and P. (P.) flavus, but it can be distinguished by the combined features of the darker brown forewing venation, the aedeagal shaft with pair of spine-like processes, and the different shape of the dorsal connective.

#### ﻿ Pediopsoides (Pediopsoides) huangi

Taxon classificationAnimaliaHemipteraCicadellidae

Li & Dai sp. nov.

DB240A4E-00AF-5505-8E15-296BA23E81D6

https://zoobank.org/7E864F85-F2CA-408B-BAEC-E3D9E3029DE1

Figs 54–60

P. (P.) femorata Huang & Viraktamath, 1993: 365; figs 18–28, misidentification.
P. (P.) huangi Li & Dai, 2018 in Dai et al. 2018: 203, figs 127A–F. Treated as a new name for P. (P.) femorata Huang & Viraktamath, 1993 (nec Pediopsisfemorata Hamilton, 1980). See Remarks.

##### Material examined.

From original figures (see Remarks). ***Holotype*** ♂, China: Taiwan, Taichung, Anmashan, 08.viii.1987, collected by C. T. Yang; ***Paratype***: 1 ♀, same data as holotype except 05.viii.1987 (both National Museum of Natural Science, Taichung, Taiwan).

##### Description.

See description by Huang and Viraktamath (1993) of P. (P.) femorata.

##### Distribution.

China (Taiwan).

##### Etymology.

The specific epithet is in honor of Dr. Kun-Wei Huang, one of the authors who originally described the species.

##### Remarks.

This species was misidentified and described as P. (P.) femorata Hamilton by Huang and Viraktamath (1993). Later it was renamed as P. (P.) huangi by Dai et al. (2018) (see Li et al. 2023) but as these authors had not indicated that it was a new species nor designated a holotype, their name had remained unavailable, and their new species was not valid. Here, we correct these omissions (see above). However unaccountably, images supposedly of the specimens described by Huang & Viraktamath, 1993, sent to the first author by Dr. Huang some years ago, do not match the habitus figures given by these authors. The latter figures show a weakly produced head compared to a greatly produced head in the received images. Unfortunately, enquiries made at the type depository (see above) indicated only an empty box was present containing a note saying that specimens had been removed for imaging. Therefore, for the present, identity of the species rests with figures of the male genitalia, reproduced here from the original (Figs 54–60).

#### ﻿ Pediopsoides (Pediopsoides) maoershanensis

Taxon classificationAnimaliaHemipteraCicadellidae

Li & Dai sp. nov.

1C4404F6-DF14-5F72-8754-104B693DE0B8

https://zoobank.org/55C08504-0D12-4CCA-84BD-8A95C7EF9D77

Figs 12–22

##### Material examined.

***Holotype*** ♂, China: Guangxi Autonomous Region, Guilin City, Maoershan National Natural Reserve, 19.vii.2015, collected by Yun-Fei Wu; ***Paratype*** 1 ♂, same data as the holotype (both SUHC).

##### Description.

***Body color*** (Figs 12–14). Ground color yellowish to dark brown with striations and punctures on surfaces of head, face, pronotum, and mesonotum pale brown. Head (Fig. 12) yellowish, anterior margin of central part weakly brown; face (Fig. 14) evenly yellowish except postclypeus with large slight brown spot centrally and anteclypeus brown to black apically; eyes brown with reddish tinge. Pronotum (Fig. 12) yellowish brown adjacent to eyes, other parts dark brown. Mesonotum (Fig. 12) yellow brown, basal triangles dark brown. Forewings (Figs 12, 13) dark brown with venation concolorous. Legs yellowish grey.

***Body morphology*** (Figs 12–14). Head including eyes (Fig. 12) narrower than pronotum, crown shorter near eyes. Face including eyes (Fig. 14) wider than long, with clear punctures and striations, postclypeus (Fig. 14) with weak longitudinal carina; distance between ocelli nearly 7.5 × that from ocellus to adjacent eye; pronotum (Fig. 12) 2.9 × broader than long, with weak transverse striations, mesonotum (Fig. 12) about 1.6 × as long as pronotum.

***Male genitalia*** (Figs 15–22). Pygofer side (Fig. 15) broad basally, relatively narrow, and prolonged caudally, ventral margin with scattered marginal setae, caudal half produced into a prominent but short spine-like process preceded by three or four spines (Fig. 16). Subgenital plate (Fig. 15) slender, slightly shorter than ventral margin of pygofer, with fine setae. Dorsal connective (Fig. 17), stout, S-shaped, axe-like, apical half wide with distinct serrated margins. Style (Fig. 18) angled at basal 1/3, stem gradually widened to apex. Connective (Figs 19, 20), anterior margin wider than posterior margin, both lateral arms twisted dorsally. Aedeagus (Figs 21, 22) simple, broad basally, bent dorsally, and gradually tapered to acute apex in lateral view, shaft of uniform width, with rounded apex in ventral view, gonopore near apex on ventral surface.

##### Measurement.

Body length (including tegmen): 4.5–4.6 mm.

##### Distribution.

China (Guangxi Autonomous Region).

##### Etymology.

The specific epithet refers to the type locality of the new species, Maoershan National Natural Reserve, combined with the Latin adjectival suffix -*ensis*, meaning from a place.

##### Remarks.

This new species is similar to P. (P.) bispinatus in appearance and coloration, and somewhat similar to P. (P.) ailaoshanensis, but can be distinguished from all members of the subgenus by the following combination of features: aedeagus simple, bent dorsally and gradually tapered to acute apex without processes; dorsal connective strongly developed, axe-like, with apical half with serrated margins. It can also be distinguished by the shape of the pygofer and pygofer processes.

#### ﻿ Pediopsoides (Pediopsoides) quadrispinosus

Taxon classificationAnimaliaHemipteraCicadellidae

Li & Dai sp. nov.

4B0175BE-4740-5B1D-853B-93EFC94A2586

https://zoobank.org/1DD04166-668A-4A8E-A25D-B37F8779973F

Figs 23–33

##### Material examined.

***Holotype*** ♂, China: Yunnan Province, Diqing Tibetan Autonomous Prefecture, Shangri-La, 08.viii.2012, collected by Zhi-Hua Fan (GUGC).

##### Description.

***Body color*** (Figs 23–25). Dorsum yellowish brown. Head (Fig. 23) yellowish with intense brown maculae; face (Fig. 23) yellowish, punctures on surface brown, postclypeus with pair of slight brown spots, below ocelli with paired spots also, eyes dark brown with reddish tinge. Pronotum (Fig. 23) yellowish brown with darker striations and punctures. Mesonotum (Fig. 23) yellow-brown with darker punctures. Forewing (Figs 23, 24) yellowish brown, several cross veins black. Legs yellow-brown with darker markings.

***Body form*** (Figs 23–25). Head including eyes (Fig. 23) almost as wide as pronotum, crown short and nearly parallel-sided, vertex clearly projecting forward angularly. Face (Fig. 25) slightly depressed in central part in lateral aspect (Fig. 24), face including eyes wider than long, surface with clear intense punctations and striae, postclypeus with distinct longitudinal carina, distance between ocelli nearly 4.3 × that from ocellus to adjacent eye. Pronotum (Fig. 23) broad, 2.3 × wider than long, with weak longitudinal carina at midlength, obliquely striated, anterior margin strongly produced forward, and posterior margin concave at midlength. Mesonotum (Fig. 23) nearly 1.2 × as long as pronotum. Forewing (Figs 23, 24) with veins prominent.

***Male genitalia*** (Figs 26–33). Pygofer side (Fig. 26) broad basally, lobe caudally truncate with dorsal and ventral margin nearly straight, apical half distinctly serrated. Subgenital plate (Fig. 26) slender, shorter than ventral margin of pygofer, surface with fine setae. Dorsal connective (Fig. 28) S-shaped, with medial long process from its ventral margin and directed posteriorly with irregular serrated margins, apex bifurcated. Style (Fig. 29) angled at basal 2/5, stem parallel-margined. Connective (Figs 30, 31), anterior margin wider than posterior margin, both lateral arms prolonged, and twisted dorsally. Aedeagus (Figs 32, 33) broad basally, shaft slender, with lateral margins sinuated in ventral view, apex with pair of short acute processes on each side of gonopore.

##### Measurement.

Body length (including tegmen): 4.4 mm.

##### Distribution.

China (Yunnan Province).

##### Etymology.

The specific epithet, *quadrispinosus*, is derived from the Latin words *quadri*- and *spinosus*, referring to the aedeagal shaft with four apical spines.

##### Remarks.

The new species is similar to P. (P.) jingdongensis in having the same yellowish brown body and body form and male pygofer ventral margin with distinct serrations in apical half formed by a row of numerous short regularly spaced denticles. It differs, however, from P. (P.) jingdongensis and all other congeners by its slender aedeagal shaft in lateral view with four apical spines and also by the shape of its dorsal connective.

#### ﻿ Pediopsoides (Pediopsoides) flavus

Taxon classificationAnimaliaHemipteraCicadellidae

Li & Dai sp. nov.

E3154D30-8C3E-54E3-8AA0-F9AD1C1B64B0

https://zoobank.org/B14A1732-D5CB-4F19-87D4-13065518435A

Figs 34–43

##### Material examined.

***Holotype*** ♂, China: Yunnan Province, Lushui City, Pianma Town, 26°0′34″N, 98°37′55″E, 1152 m above sea level, 26.v.2019, collected by Jia-Jia Wang and Chao Zhang; ***Paratypes***: 1 ♂ 1 ♀, same data as the holotype (GUGC).

##### Description.

***Body color*** (Figs 34–36). Head (Fig. 34) lemon yellow, central part slightly brownish yellow; face (Fig. 36) evenly lemon yellow except for slightly brownish yellow postclypeus, eyes black. Pronotum (Fig. 34) with yellow ground color, slightly brown to dark brown or even black around median line and posterior margin. Mesonotum (Fig. 34) yellowish brown, basal triangles black, punctations on surface dark brown, posterior submargin of mesoscutum with pair of small black spots. Forewing (Figs 34, 35) dark brown, veins concolorous. Legs evenly yellowish.

***Body form*** (Figs 34–36). Head including eyes (Fig. 34) narrower than pronotum, crown very short, roundly projecting forward. Face (Fig. 36) including eyes slightly wider than long, postclypeus with indistinct longitudinal carina, distance between ocelli nearly 6 × that from ocellus to adjacent eye. Pronotum (Fig. 34) 2.6 × wider than long, with weak oblique striations, anterior margin projecting forward, and posterior margin clearly concave at midlength. Mesonotum (Fig. 34) nearly 1.68 × longer than pronotum. Forewing (Figs 34, 35) with veins prominent.

***Male genitalia*** (Figs 37–43). Pygofer side (Fig. 37) broad basally, lobe with caudal margin truncate and dorsal margins straight, ventral margin with apical half margined with several minute teeth (Fig. 38) and marginal setae. Subgenital plate (Fig. 37) slender, nearly parallel sided, distinctly shorter than ventral margin of pygofer, surface with fine setae. Dorsal connective (Fig. 39) with apical process long, tapered gradually, and twisted basally with acute tip, lateral margins smooth. Style (Fig. 40) slightly angled at basal 2/5, stem slightly sinuate, and slightly broader in central part. Connective (Fig. 41) with anterior margin wider than posterior margin, both lateral arms twisted dorsally. Aedeagus (Figs 42, 43) broad basally, dorsal apodeme developed, shaft slender, bent dorsally, tapered to pointed apex and U-shaped in lateral view, in ventral aspect shaft with round apex, inflated at midlength, lateral margins with pair of small spines pointed basolaterally, gonopore apical on ventral surface.

##### Measurement.

Body length (including tegmen): 5.5–5.6 mm.

##### Distribution.

China (Yunnan Province).

##### Etymology.

The specific epithet is derived from the Latin word *flavus*, referring to the yellowish color of the species especially on the face.

##### Remarks.

The new species is similar to P. (P.) damingshanensis and P. (P.) ailaoshanensis but can be distinguished from them and other congeners by the following male genitalia characters: aedeagus with shaft inflated at midlength in ventral view with a pair of small spines near apex; pygofer ventral margin with apical half distinctly margined with several minute teeth; differently shaped dorsal connective.

#### ﻿ Pediopsoides (Pediopsoides) pianmaensis

Taxon classificationAnimaliaHemipteraCicadellidae

Li & Dai sp. nov.

ED7337B6-1BB0-5EFB-906E-44FF01E6367C

https://zoobank.org/D01764DB-3185-4000-8A99-41387246293A

Figs 44–53

##### Material examined.

***Holotype*** ♂, China: Yunnan Province, Lushui City, Pianma Town, 26°0′34″N, 98°37′55″E, 1152 m above sea level, 26.v.2019, collected by Jia-Jia Wang and Chao Zhang; ***Paratype***: 1 ♂, same data as the holotype (GUGC).

##### Description.

***Body color*** (Figs 44–46). Head yellowish brown; face (Fig. 46) with postclypeus with large pale brown area centrally, anteclypeus with black apical half, eyes brown to black with reddish tinge. Pronotum (Fig. 44) yellowish brown on regions near eyes and lateral margins, other parts dark brown to black. Mesonotum (Fig. 44) yellowish brown, basal triangles and two small round spots between them black, median stripe dark brown. Forewing (Figs 44, 45) brown, veins darker. Legs yellowish brown with black marks.

***Body form*** (Figs 44–46). Head across eyes (Fig. 44) narrower than pronotum, crown short, parallel sided. Face across eyes (Fig. 46) wider than long, surface with clear punctures and striae, upper part of postclypeus with weak longitudinal carina, distance between ocelli nearly 6.2 × that from ocellus to adjacent eye. Pronotum (Fig. 44) with indistinct longitudinal carina medially, 2.9 × as wide as long, with weak oblique striations, anterior margin roundly produced in front of eyes and posterior margin concave at midlength. Mesonotum (Fig. 44) ~ 1.7 × as long as pronotum, triangular. Forewing (Figs 44, 45) with venation prominent.

**Figures 61–82.**
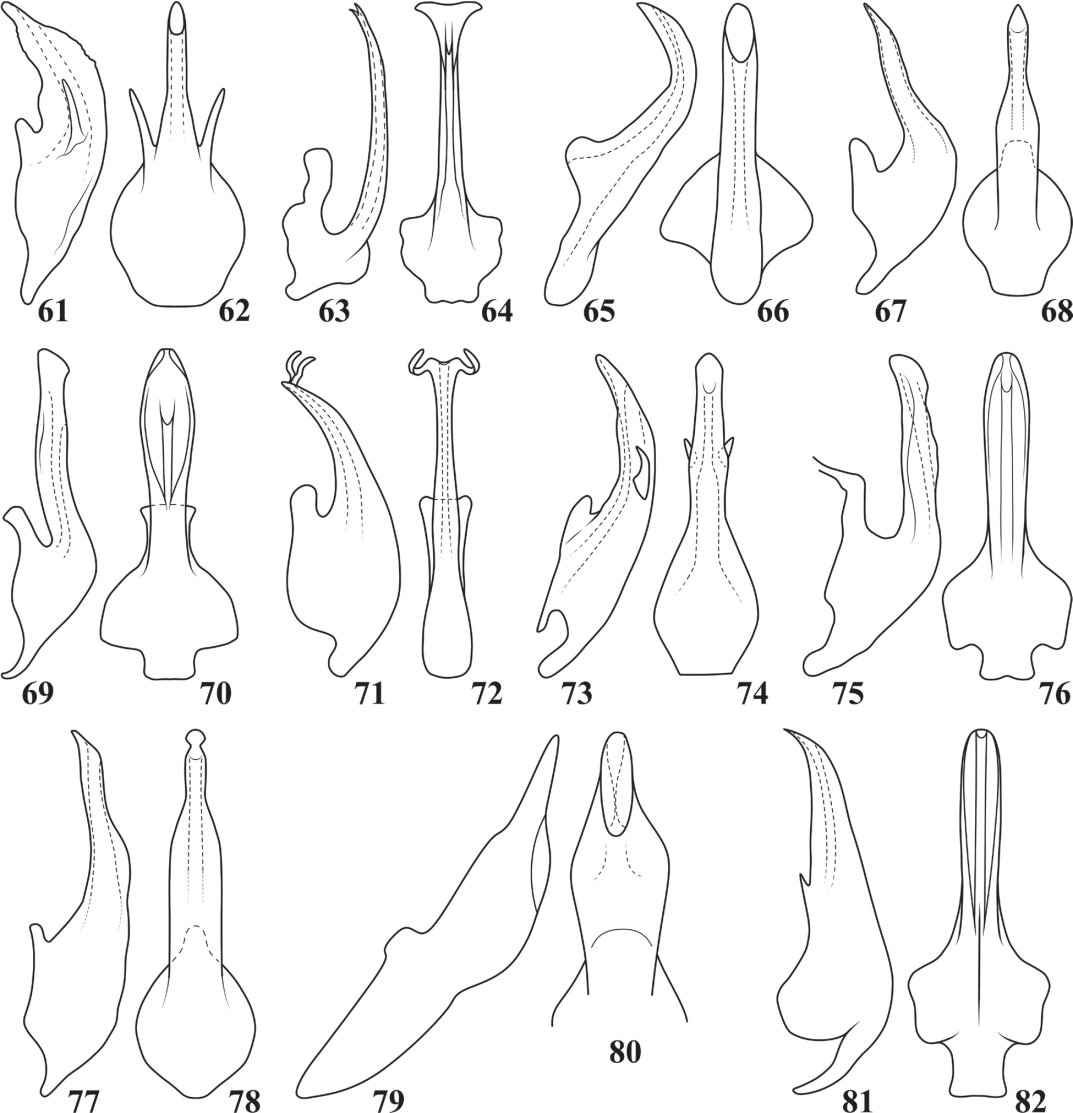
Aedeagus of Pediopsoides (Pediopsoides) species, lateral (61, 63, 65, 67, 69, 71, 73, 75, 77, 79, 81) and ventral view (62, 64, 66, 68, 70, 72, 74, 76, 78, 80, 82) **61, 62**P. (P.) albus**63, 64**P. (P.) amplificatus**65, 66**P. (P.) anchorides (after Yang and Zhang 2013) **67, 68**P. (P.) bispinatus**69, 70**P. (P.) damingshanensis**71, 72**P. (P.) jingdongensis (after Zhang 2010) **73, 74**P. (P.) kodaiana (after Viraktamath 1996) **75, 76**P. (P.) longiapophysis**77, 78**P. (P.) nigrolabium**79, 80**P. (P.) satsumensis (after Hamilton 1980) **81, 82**P. (P.) tishetshkini.

***Male genitalia*** (Figs 47–53). Pygofer side (Fig. 47) broad basally, lobe stout, slightly prolonged caudally, with dorsal margin straight with rounded caudal margin, ventral margin infolded, caudal half with prominent tooth-like distal process preceded by a series of small teeth (Fig. 48). Subgenital plate (Fig. 47) shorter than ventral margin of pygofer, surface with fine setae. Dorsal connective as in Fig. 49. Style (Fig. 50) angled at about basal 2/5, stem slightly broader in central part and slightly narrowed subapically. Connective (Fig. 51), anterior margin wider than posterior margin, both lateral arms twisted dorsally. Aedeagus (Figs 52, 53) basally broad, dorsal apodeme developed; in lateral aspect, shaft gradually tapered to acute apex, bent dorsally, nearly U-shaped, in ventral view, weakly tumid at midlength, apex rounded, without process, gonopore apical.

##### Measurement.

Body length (including tegmen): 4.6–4.7 mm.

##### Distribution.

China (Yunnan Province).

##### Etymology.

The specific epithet refers to the type locality of the new species, Pianma Town, combined with the Latin adjectival suffix -*ensis*, meaning from a place.

##### Remarks.

The new species has the dorsal connective similar to that in P. (P.) ailaoshanensis, but can be distinguished from the latter by the simple aedeagal shaft without processes; it differs from other congeners by the combined features of dorsal connective, aedeagus and male pygofer processes.

## Supplementary Material

XML Treatment for Pediopsoides (Pediopsoides)

XML Treatment for Pediopsoides (Pediopsoides) ailaoshanensis

XML Treatment for Pediopsoides (Pediopsoides) huangi

XML Treatment for Pediopsoides (Pediopsoides) maoershanensis

XML Treatment for Pediopsoides (Pediopsoides) quadrispinosus

XML Treatment for Pediopsoides (Pediopsoides) flavus

XML Treatment for Pediopsoides (Pediopsoides) pianmaensis

## ﻿Additional information

### Author ORCIDs

Hu Li https://orcid.org/0000-0002-5453-6084

Juan Li https://orcid.org/0009-0005-1899-6621

Michael D. Webb https://orcid.org/0000-0002-1312-6142

Jia-Jia Wang https://orcid.org/0000-0002-1843-3977

Ren-Huai Dai https://orcid.org/0000-0001-7652-6808

### Data availability

All of the data that support the findings of this study are available in the main text.
